# Architecture and hydration of the arginine‐binding site of neuropilin‐1

**DOI:** 10.1111/febs.14405

**Published:** 2018-02-25

**Authors:** Filipa Mota, Constantina Fotinou, Rohini R. Rana, A. W. Edith Chan, Tamas Yelland, Mohamed T. Arooz, Andrew P. O'Leary, Jennie Hutton, Paul Frankel, Ian Zachary, David Selwood, Snezana Djordjevic

**Affiliations:** ^1^ Magnus Life Magnus Life Science London UK; ^2^ Wolfson Institute for Biomedical Research University College London UK; ^3^ The Institute of Structural and Molecular Biology University College London UK; ^4^ Centre for Cardiovascular Biology & Medicine BHF Laboratories at University College London UK; ^5^ Present address: Department of Imaging Sciences King's College London St. Thomas's Hospital London SE1 7EH UK

**Keywords:** ligand‐binding protein, neuropilin, SPR, vascular endothelial growth factor (VEGF), X‐ray crystallography

## Abstract

Neuropilin‐1 (NRP1) is a transmembrane co‐receptor involved in binding interactions with variety of ligands and receptors, including receptor tyrosine kinases. Expression of NRP1 in several cancers correlates with cancer stages and poor prognosis. Thus, NRP1 has been considered a therapeutic target and is the focus of multiple drug discovery initiatives. Vascular endothelial growth factor (VEGF) binds to the b1 domain of NRP1 through interactions between the C‐terminal arginine of VEGF and residues in the NRP1‐binding site including Tyr297, Tyr353, Asp320, Ser346 and Thr349. We obtained several complexes of the synthetic ligands and the NRP1‐b1 domain and used X‐ray crystallography and computational methods to analyse atomic details and hydration profile of this binding site. We observed side chain flexibility for Tyr297 and Asp320 in the six new high‐resolution crystal structures of arginine analogues bound to NRP1. In addition, we identified conserved water molecules in binding site regions which can be targeted for drug design. The computational prediction of the VEGF ligand‐binding site hydration map of NRP1 was in agreement with the experimentally derived, conserved hydration structure. Displacement of certain conserved water molecules by a ligand's functional groups may contribute to binding affinity, whilst other water molecules perform as protein–ligand bridges. Our report provides a comprehensive description of the binding site for the peptidic ligands’ C‐terminal arginines in the b1 domain of NRP1, highlights the importance of conserved structural waters in drug design and validates the utility of the computational hydration map prediction method in the context of neuropilin.

**Database:**

The structures were deposited to the PDB with accession numbers PDB ID: 5IJR, 5IYY, 5JHK, 5J1X, 5JGQ, 5JGI.

**Abbreviations**
NRP1: Neuropilin‐1
NRP1‐b1: B1 domain of Neuropilin‐1
NRPs: Neuropilin‐1 and Neuropilin‐2
PDB: protein data bank
VEGF‐A_165_: vascular endothelial growth factor

## Introduction

Neuropilins (NRPs) are transmembrane receptors that take part in protein–protein interactions, and are essential for formation of the mammalian nervous and vascular systems 1, 2. NRPs were initially discovered as neuronal receptors for class‐3 semaphorins and mediators of axonal guidance in the developing nervous system 3. NRPs also bind a number of growth factors, including vascular endothelial growth factors (VEGFs) 4, 5, transforming growth factor (TGF‐β) 6, 7 and hepatocyte growth factor (HGF) 8. Furthermore, NRPs are involved in the Hedgehog signal transduction pathway 9, and tumour immune regulation through regulatory T cells 10. Two homologous proteins, neuropilin‐1 (NRP1) and neuropilin‐2 (NRP2), share 44% sequence identity and a similar multi‐domain structure. The interaction of NRP1 with VEGF‐A_165_ has been extensively studied, and is implicated in the processes of angiogenesis 11, cell migration and metastasis 12, and evasion of an immune response in cancer 13, 14. VEGF‐A_165_ binds to a well‐defined binding site on the b1 domain of NRP1 (NRP1‐b1) through its C‐terminal arginine residue. It is possible that other ligands containing a C‐terminal arginine will also bind in a similar fashion 15. Although protein–protein interactions have been considered challenging targets in drug discovery 16, a number of small molecules and peptides have been identified as inhibitors of the VEGF‐A_165_–NRP1 interaction 17, 18, 19, 20, 21, 22, 23, 24, 25, 26, 27, 28, 29. In some cases, it has been demonstrated that the inhibitors act through direct binding to the b1 domain of NRP1. These inhibitors, exemplified by EG00229, also incorporate an arginine residue and bind at the same site as the C‐terminus of VEGF‐A_165_ 19.

We have conducted an extensive study of the C‐terminal arginine‐binding site in NRP1‐b1, focusing on protein side chain flexibility and conserved water molecules. Hydration of proteins is an important parameter to consider in drug design. Structural waters may play a key role in the function, shape and conformation of proteins, as well as in protein–ligand interactions 30, 31. The incorporation of structural waters in computational drug design may significantly improve the outcome of molecular docking results 32, 33, 34. Water molecules can act as bridges between the protein‐binding site and a ligand or cofactor via hydrogen bonds 32, 33, 34, 35. Disruption of these water networks by small molecule compounds may have an effect on the binding affinity of such molecules. Replacement of a tightly bound water molecule does not, however, necessarily improve binding affinity or ligand efficiency, and it may influence the pharmacodynamic profile of the molecule 32, 33, 34, 35. It is therefore important to consider structural or conserved waters in the binding site, and to explore their role in ligand‐binding efficiency either by replacing them with functional groups or by incorporating them as hydrogen‐bond bridges between the ligand and the protein. Unfortunately, due to poor structural understanding or due to lack of availability of this information from protein crystal structures (e.g. in low‐resolution structures), water molecules are often not included in structure‐based drug design. As an alternative, computational models may be used to predict the location of water molecules in protein structures 34, 36.

We selected a number of arginine analogues with simple structural variations to perform this study, and obtained high‐resolution structures of six compounds bound to NRP1‐b1. Surface plasmon resonance (SPR) was used to determine the dissociation constant (*K*_D_) of the arginine analogues to immobilised NRP1‐b1. The binding data, together with computational methods and crystallographic information, was used to rationalise the side chain flexibility and hydration of the binding site in NRP1‐b1. The new crystal structures were analysed alongside publicly available NRP1‐b1 structures in the protein data bank (PDB). This comprehensive analysis and knowledge of the detailed architecture of the VEGF‐A_165_ binding site in NRP1‐b1 will facilitate future drug design efforts.

## Results and Discussion

### Prediction of binding site hydration structure

**Figure 1:**
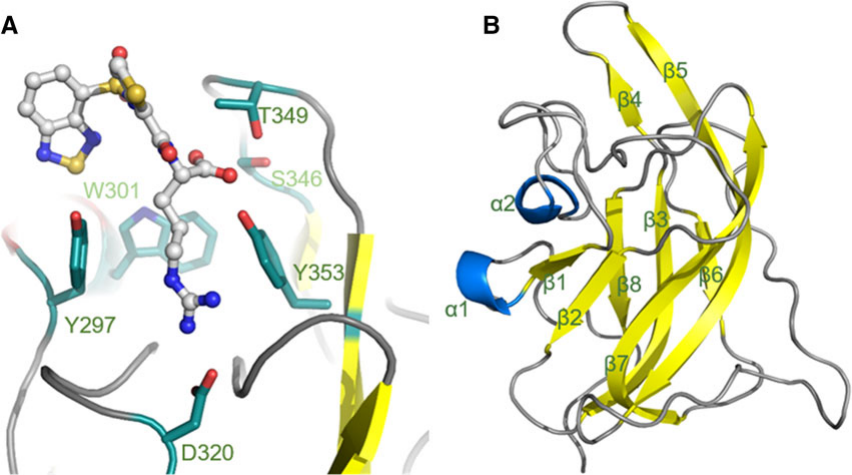
Structure of the binding site of NRP1‐b1 domain. (A) Ball and stick representation of EG00229 (carbon atoms are coloured grey, nitrogen blue, sulphur yellow and oxygen red) bound to NRP‐b1 (PDB entry 3I97). NRP1‐b1 domain residues involved in the non‐covalent interactions with the ligand are shown in sticks representation. (B) Ribbon diagram of NRP1‐b1 fold: β‐sheets are represented in yellow and α‐helixes in blue.

**Figure 2:**
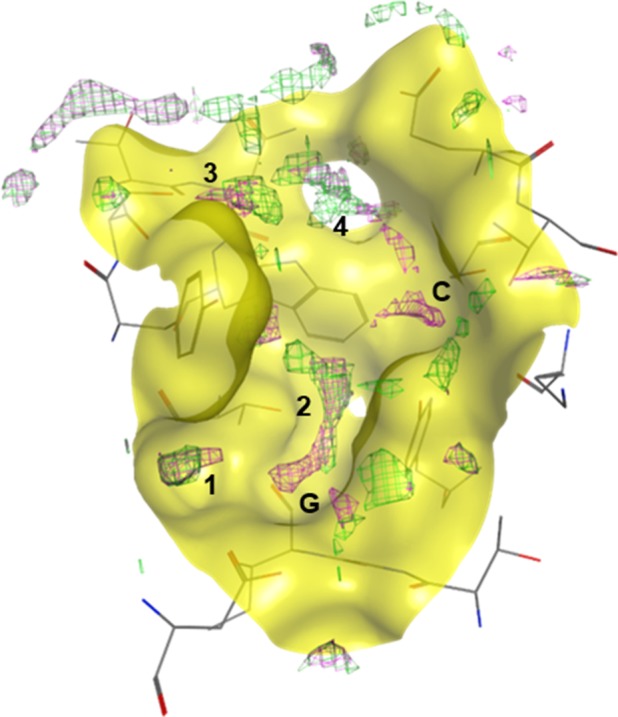
Prediction model of NRP1‐b1 binding site hydration. Computational analysis performed on PDB entry 3I97, where the green mesh represents the solvation prediction with EG00229 bound to NRP1‐b1, and the pink mesh represents the solvation prediction of NRP1‐b1 in the apo form. Labels **1–4** indicate the regions predicted to be occupied by water molecules in the binding site; **G** and **C** indicate the regions occupied by the arginine guanidine and carboxylate groups, respectively, if EG000229 is bound. In the apo form of NRP1‐b1, these regions are also predicted to be occupied by water molecules.

We initiated our study into the binding site hydration structure of NRP1‐b1 domain by predicting the position of water molecules using computational models. We used a solvent analysis application within the software package MOE (Chemical Computing Group), which assesses the role of solvent in protein structures, using a three‐dimensional reference interaction site model (3D‐RISM). This method allows for the calculation of the solvation structure represented by the probability density function of finding interaction sites for the solvent molecules (in this case, water molecules) at the specific point around the solute molecule (in this case, our protein target NRP1‐b1) 34, 35, 36, 37. At the time this study was designed, there were 11 structures of NRP1 deposited in the PDB, 7 of which are of human origin (PDB codes: 4RN5, 4DEQ, 3I97, 2QQI, 2QQM, 2QQN, 1KEX), 2 mouse (PDB codes: 4GZ9, 4GZA) and 2 rat (PDB codes: 2ORX, 2ORZ). These structures corresponded either to the isolated b1 domain or tandem b1b2 and a2b1b2 domains of NRP1. The structure of EG00229‐bound NRP1‐b1 (PDB code: 3I97) was selected for initial computational studies (Fig. 1). Although this was not the structure of highest resolution available at the time, it was the only one with a synthetic ligand present at the binding site, and thus deemed appropriate for analysing the effect of ligand‐binding on the solvation structure of the receptor protein. The calculations were performed on the protein–ligand complex, the protein alone with the ligand excluded from the calculations and on the ligand alone. To visualise these calculations, the predicted distribution of water density was plotted on the binding site of NRP1‐b1 (Fig. 2). There is a clear overlap of the distribution of the predicted water density between the ligand‐bound and the ligand‐free protein state. However, the comparison of the predicted solvation distribution in the presence and absence of ligand shows that guanidine and carboxylic acid groups of EG00229 occupy two of the distinct patches of the solvation space predicted to exist in the absence of the ligand (regions G and C in Fig. 2). The predictions thus suggest that ligand binding is accompanied by solvent‐displacement. In addition to the areas of the molecular surface occupied by the guanidine and the carboxylate groups, four other regions, labelled 1**–**4, show water density in the binding site identified from the calculations performed with either the protein alone or NRP1‐b1‐EG00229 complex (Fig. 2).

### Selection of ligands for the study

**Figure 3:**
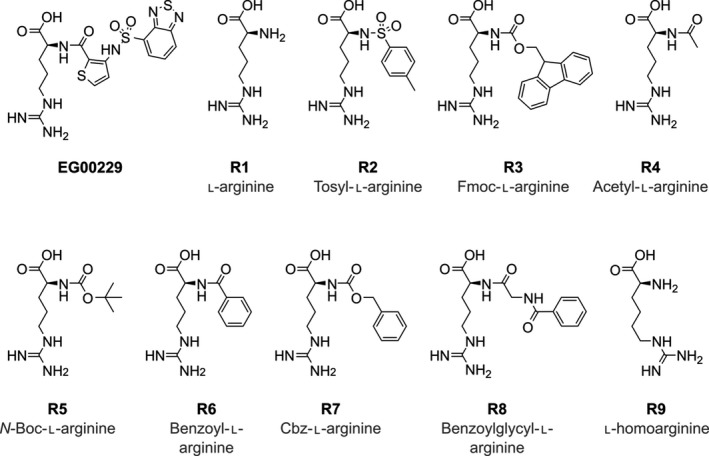
Chemical structures of selected arginine analogues **R1–R9.**

EG00229 is a small molecule formed of an arginine residue with *N*α‐substitution. Key interactions of EG00229 with NRP1‐b1 are established through hydrogen bonds between the guanidine and carboxylic acid groups of the ligand to the receptor. To gain more comprehensive understanding of the ligand‐binding site structure which would assist us in the design of NRP1 antagonists, we investigated interaction between NRP1‐b1 domain and multiple commercially available arginine analogues. The ligands selected for this study retain the arginine moiety as seen in EG00229, while providing structural diversity in the *N*α‐site of the molecule (Fig. 3). l‐arginine (**R1**) and seven commercially available *N*α‐substituted‐l‐arginine analogues **R2–R8** were selected to explore their binding affinities and the solvation profile of the NRP1‐b1 ligand‐binding site experimentally. In addition, underivatised l‐homoarginine (**R9**) was also included in the study (Fig. 3). As we were only interested in probing the binding site and the crystallographic behaviour of the ligands, metabolic stability of the compounds was not considered. Thus, we were able to probe the binding site with a relatively simple, readily available and cheap set of analogues.

A surface plasmon resonance (SPR) method was used to determine the binding affinities of the ligands to NRP1‐b1, and crystallisation trials were performed with the preformed complexes between NRP1‐b1 and the individual ligands.

### Ligand‐binding affinities

**Figure 4:**
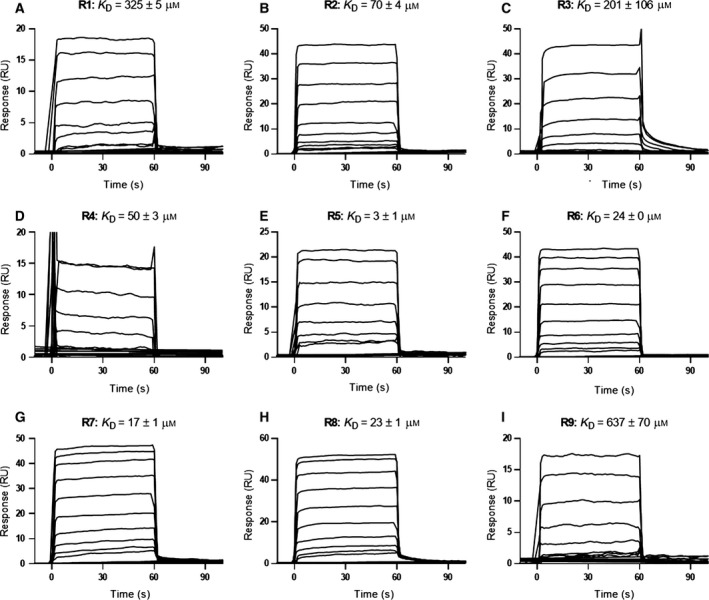
SPR measurements of arginine analogues **R1–R9** binding to immobilised NRP1‐b1. Equilibrium dissociation constants (*K*_D_) were calculated using steady‐state binding levels and assuming a 1 : 1 binding model of the arginine analogues **R1–R9** to immobilised NRP1‐b1. All sensorgrams are double‐referenced, using a blank surface and sample. Concentration ranges are as follows: **R1** and **R9** were tested at 12**–**1500 μm,** R5** was tested at 0.16**–**20 μm, all other analogues were tested at 0.6**–**300 μm (*n* = 2).

All arginine analogues showed very fast rates of association and dissociation to immobilised NRP1‐b1, as determined by surface plasmon resonance experiments (Fig. 4). Binding responses were consistent with a 1 : 1 binding interaction model and equilibrium binding affinities were determined using steady‐state binding levels to afford dissociation constants (Fig. 4). l‐Arginine **R1** and l‐homoarginine **R9** showed weak binding to NRP1‐b1, with dissociation constants (*K*_D_s) of 325 ± 5 and 637 ± 70 μm, respectively. These are the weakest binding ligands tested, with all other *N*α‐substituted analogues exhibiting higher affinities and lower corresponding dissociation constants. The data suggest that the free amine is not favourable for binding, and that the longer alkane chain on l‐homoarginine might de‐stabilise key interactions with the receptor. Interestingly, a large *N*α substituent, as seen in FMOC‐l‐arginine (**R3**) also resulted in a weakly binding ligand, with a *K*_D_ of 201 ± 106 μm; however, this molecule's poor solubility might have affected the results. Tosyl *N*α‐substituted l‐arginine (**R2),** acetyl‐l‐arginine (**R4**), benzoyl‐l‐arginine (**R6**) and benzoylglycyl‐l‐arginine (**R8**) exhibited a medium range of affinities with *K*_D_s between 70 and 22 μm (Fig. 4) without a clear structure/activity relationship. The two other analogues containing carbamate groups, N‐boc‐l‐arginine (**R5**) and Cbz‐l‐arginine (**R7**) exhibited highest affinities for NRP1‐b1 in the series, with *K*_D_s of 3 ± 1 and 17 ± 1 μm, respectively.

**Figure 5:**
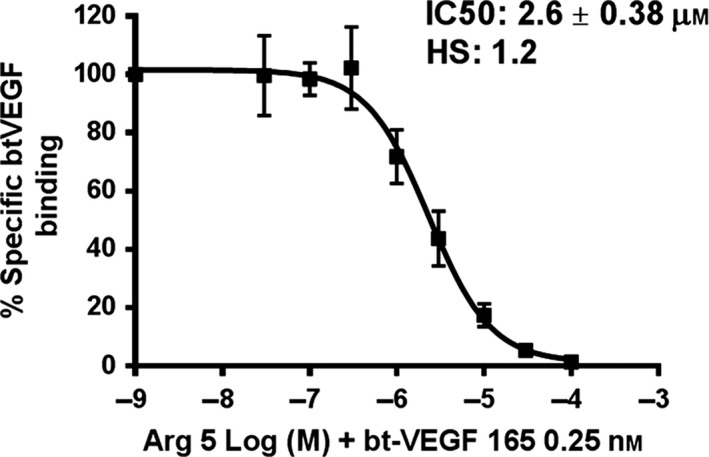
Inhibition of bt‐VEGF‐A165 binding to NRP1 by R5 arginine analogue**.** The various concentrations of **R5** compound were added to the 96‐well plates precoated with NRP1‐b1 protein, followed by addition of 0.25 nm bt‐VEGF‐A165. Non‐specific binding of bt‐VEGF‐A165 to the plates was determined in the absence of NRP1‐b1. **R5** analogue competed with bt‐VEGF‐A165 for binding to plates coated with NRP1 with an IC50 of 2.6 μm. Values presented are the means ± SEM obtained from three independent experiments each performed in duplicates.

The analogues were also tested in a ligand displacement essay assessing their potency to compete with the VEGF‐A_165_ for binding to NRP1‐b1. **R5**, the Arg analogue with the highest affinity in the SPR assay, exhibited clear sigmoidal curve in a ligand displacement essay with an IC50 of 2.6 ± 0.38 μm, which correlates extremely well with the SPR data (Fig. 5). However, while **R6**,** R7** and **R8** exhibited weak activity, we were unable to reproducibly determine their IC50 values as the binding curves did not display sigmoidal features.

### Crystal structures of the NRP1‐b1 domain complexed with arginine analogues

Co‐crystallisation studies were attempted with all ligands selected and six complexes produced good‐quality crystals. All six complexes crystallised in the monoclinc (P2_1_) space group with a dimer in the asymmetric unit for all complexes except for that of **R5** analogue which contained a tetramer in the asymmetric unit. The structures were refined using data at the resolutions higher than 2.1 Å (for **R5**‐bound NRP1 b1); full data and refinement statistics are available in Table 1.

**Table 1:** Data collection and refinement statistics. The values in parentheses are for the last resolution shell

Ligand	R4	R5	R6	R7	R8	R9
PDB ID	5JGI	5J1X	5JGQ	5IYY	5JHK	5IJR
Data collection						
Space group	P 21	P 21	P 21	P 21	P 21	P 21
Unit cell parameters (Å)	40.65	41.59	40.79	41.52	40.38	40.14
	89.20	90.48	89.57	88.94	90.78	88.94
	41.65	81.89	41.38	41.70	40.97	40.96
β (°)	98.71	99.64	98.57	91.61	98.27	96.73
Molecules per asymmetric unit	2	4	2	2	2	2
Resolution range (Å)^a^	1.38 (1.40–1.38)	2.10 (2.16–2.10)	1.60 (1.63–1.60)	1.60 (1.63–1.60)	1.80 (1.84–1.80)	1.52 (1.55–1.52)
Rmerge	0.035 (0.136)	0.103 (0.476)	0.047 (0.442)	0.117 (0.436)	0.058 (0.552)	0.048 (0.510)
CC(1/2)	0.999 (0.987)	0.995 (0.820)	0.998 (0.844)	0.994 (0.871)	0.999 (0.872)	1.00 (0.866)
<I/σ (I)>	28.4 (8.6)	9.1 (2.6)	10.5 (2.0)	9.9 (3.5)	22.6 (3.2)	22.8 (2.8)
Completeness (%)	95.7 (70.0)	99.8 (99.8)	97.0 (75.3)	99.7 (99.0)	99.9 (99.9)	95.4 (67.8)
Number of total reflections	377 819 (10823)	130 425 (10307)	115 306 (2763)	270 340 (12410)	181 428 (10898)	276 749 (7499)
Number of unique reflections	57 512 (2073)	34 913 (2843)	37 578 (1441)	39 753 (1908)	27 093 (1632)	41 807 (1464)
Multiplicity	6.6 (5.2)	3.7 (3.6)	3.1 (1.9)	6.8 (6.5)	6.7 (6.7)	6.6 (5.1)
Refinement statistics						
Rwork/Rfree (%)	15.10/16.91	18.16/22.32	16.05/19.30	14.8/18.7	16.74/21.43	18.11/21.29
Mean B factor (Å^2^)	13.705	22.39	19.59	13.42	24.74	20.08
R.m.s deviations from ideal						
Bond lengths (Å)	0.020	0.013	0.020	0.023	0.016	0.018
Bond angles (°)	1.978	1.517	1.864	2.117	1.732	1.883
Ramachadran plot (%)						
Residues in favoured region	94.28	92.88	93.33	93.36	92.51	95.05
Residues in allowed region	4.38	5.79	5.33	5.32	6.19	3.63
Residues in disallowed region	1.35	1.32	1.33	1.33	1.30	1.32

a The values in parentheses correspond to the highest resolution shell.

**Figure 6:**
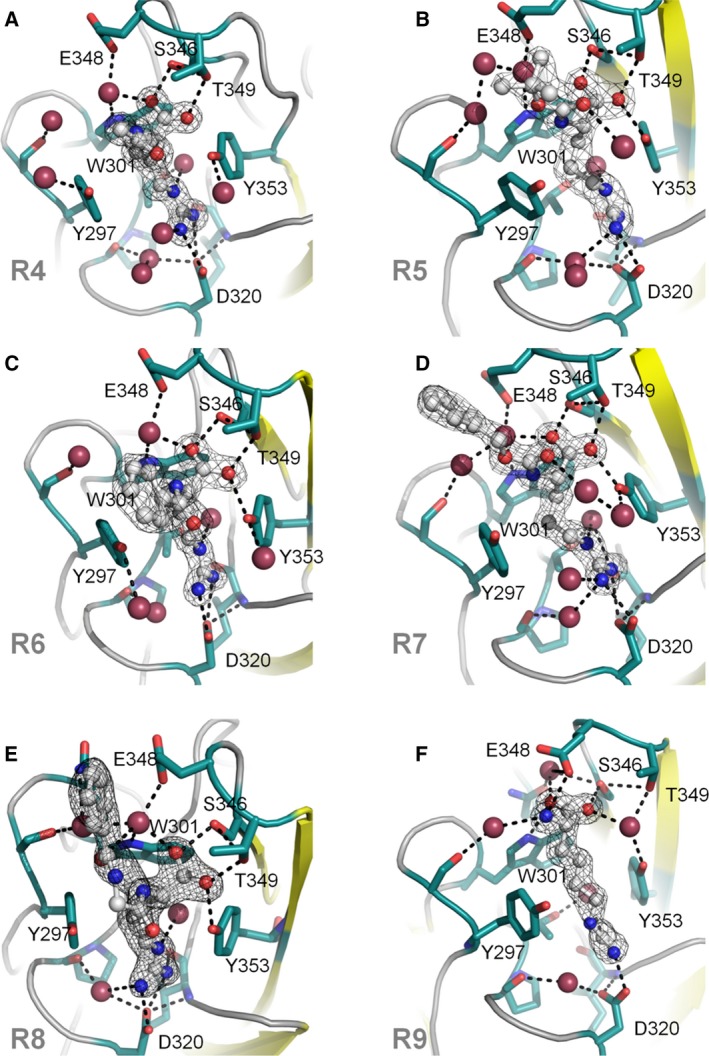
X‐ray crystal structures of arginine analogues R4**–**R9 bound to NRP1‐b1. Side chains of key residues in the binding site are shown in green, ligands are shown in grey, nitrogen atoms are shown in blue and oxygen atoms are shown in red. Oxygen atoms corresponding to water molecules are larger and shown in raspberry colour. The figures were generated in PyMOL. The electron density of the 2Fo‐Fc map is shown as a wire mesh and contoured to 1 sigma level; hydrogen bonds are represented as black dashes. (A) **R4** (PDB entry 5JGI); (B) **R5** (PDB entry 5J1X); (C) **R6** (PDB entry 5JGQ); (D) **R7** (PDB entry 5IYY); (E) **R8** (PDB entry 5JHK); (F) **R9** (PDB entry 5IJR).

The NRP1‐b1 domain belongs to the discoidin structural domain family that also includes homologous FV/VIII C domains. The fold in this type of domain is characterised by an eight‐stranded distorted jellyroll β‐barrel where a five‐stranded antiparallel β‐sheet packs against a three‐stranded antiparallel β‐sheet. The VEGF‐A_165_ binding site is located at the top of the β‐barrel core delineated by six juxtaposed loops connecting the β‐strands (Fig. 1B). All arginine analogues were found bound at the same site and are positioned in a similar way as the arginine moiety of EG00229 and the terminal arginine of VEGF‐A_165_ within the binding site of NRP1‐b1 (Figs 1 and 6). While in the crystal structures of the complexes containing ligands **R4**,** R6**,** R8** and **R9**, clearly interpretable electron density was found in only one copy of the b1 domain within the dimeric asymmetric unit, in the structures of the complexes of the higher affinity ligands (**R5** and **R7**), we were able to identify the arginine analogue bound to each of the b1 molecules within the asymmetric unit of the respective unit cells. In the structure of the complexes with the ligands **R4–R8**, the side chain guanidine group forms bidentate hydrogen bonds with Asp320. The aliphatic part of arginine is located in the groove between Tyr297 and Tyr353, with a conformation favouring a π–π stacking between the arginine side chain and the phenyl rings. The carboxylate group anchors the small molecules specifically through hydrogen‐bond interactions such that one of the oxygen atoms binds to the hydroxyl of Ser346, while the other carboxylate oxygen forms hydrogen bonds with the hydroxyl oxygen atoms from Thr349, and Tyr353. These interactions are analogous to that observed for the C‐terminus of the peptide ligands 38, 39 and are also conserved in a structure of acetate bound to NRP1‐b1 (PDB code: 4RN5).

Among the obtained crystal structures of the complexes, **R9** is the only analogue without the *N*α‐substituent, and with an extra methylene group in the side chain when compared to the natural amino acid arginine. Longer aliphatic chain affected a shift in the positioning of the carboxyl group such that it binds differently to all other ligands in the ligand‐bound structures presented here. Compared to other ligands, the carboxylate of **R9** is moved by more than 2 Å towards the protein core such that one of the oxygen atoms interacts directly with the hydroxyl from Ser346, whereas the other oxygen atom forms direct hydrogen bond with the nitrogen atom from the side chain of the residue Trp301. Furthermore, the guanidine group now forms single hydrogen bond with Asp320 compared to bidentate interaction observed in the structures of the b1 complexes with the other arginine analogues. Although the free amino group of **R9** engages in a hydrogen bond with Glu348, the total number of direct hydrogen bonds to the protein chain is four compared to five observed with the other analogues. This shift in binding mode is accompanied by a significant decrease in binding affinity, as shown by SPR.

**Figure 7:**
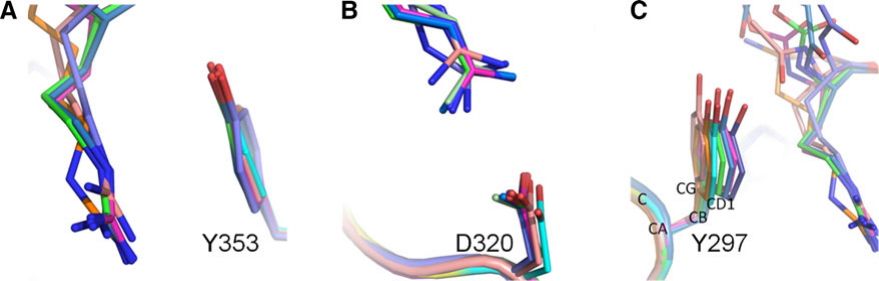
Side chain flexibility of Asp320 and Tyr297 in the NRP1‐b1 binding site. Effect of ligand binding on the side chain conformation of Y297, Y353 and D320 amino acids. The complexed protein structures (pdb IDs: 5JGI – dark pink, 5J1X – dark blue, 5JGQ – lilac, 5IYY – light pink, 5JHK – green, 5IJR – orange) were superimposed over the apo‐structure of NRP1‐b1 domain (pdb code: 1KEX, turquoise colour) (A) π–π stacking between the guanidino‐group of the bound small molecules and phenyl ring of Y353 residue. (B) A stick representation of the side chain rotamers of D320 residue of NRP1‐b1 protein as it has been observed in the X‐ray structures of the complexes. The interacting portion of the arginine analogues is shown as well. (C) A demonstration of the variability of the rotamer conformation of Y297 amino acid upon compound binding. As it is shown in Table 2, the differences between the values of torsion angles Chi1 and Chi2 of the ligand‐bound structures and those in the apo structure are significant and vary depending on the compound.

Overall, the protein components of the six arginine analogue‐bound structures show high structural similarity, with only discernible conformational differences evident in an area of the b1 domain engaged in forming the intermolecular interaction within the dimer/tetramer of the asymmetric unit in the bound structures and the side chains of residues Tyr297 and Asp320 in the ligand‐binding site. The two side‐chain differences reflect the structural differences of the bound arginine analogues. The differences are best depicted by the changes in the torsion angle of the Y297 side chain as shown in Fig. 7 and Table 2.

**Table 2:** Side chain flexibility of D320 and Y297 in the NRP1‐b1‐binding site

PDB ID	Ligand	Y297Chi1	Y297Chi2	D320Chi1	D320Chi2
1KEX (apo)		−167.7	67.6	−69.1	−37.9
5IJR, chain A	R4	−178.3	−88.1	−58.3	−55.1
5J1X, chain A	R5	−173.8	−80.6	−63.5	−44.0
5J1X, chain B	R5	−172.6	83.0	−62.2	−41.9
5J1X, chain C	R5	−173.7	84.1	−61.4	−41.8
5J1X, chain D	R5	−175.1	83.6	−61.4	−41.5
5JGQ, chain B	R6	179.3	74.4	−61.6	−54.9
5IYY, chain A	R7	−175.7	93.6	−59.5	−55.9
5IYY, chain B	R7	−178.3	86.6	−61.2	−51.0
6JHK, chain A	R8	−171.9	−88.3	−62.9	−51.3
5JGI, chain B	R9	−167.9	79.7	−59.7	−47.4

### Analysis of ordered water molecules

The high‐resolution X‐ray crystal structures showed additional density consistent with water molecules in the binding site. To conduct an analysis of the conserved water molecules in the binding site of NRP1‐b1, we considered all new structures reported here together with the already publicly available structures. Only structures of human NRP1 with a resolution higher than 2.1 Å were selected for analysis. Table 3 summarises the selected X‐ray structures.

**Table 3:** Selected X‐ray crystal structures for binding site hydration studies

PDB code	Resolution (Å)	Protein domain	Ligand in binding site
2QQI	1.80	b1b2	(apo)
4RN5	1.70	b1	Acetate ion
1KEX	1.90	b1	(apo)
5JGI	1.38	b1	R4
5J1X	2.10	b1	R5
5JGQ	1.60	b1	R6
5IYY	1.60	b1	R7
6JHK	1.80	b1	R8
5IJR	1.52	b1	R9

**Figure 8:**
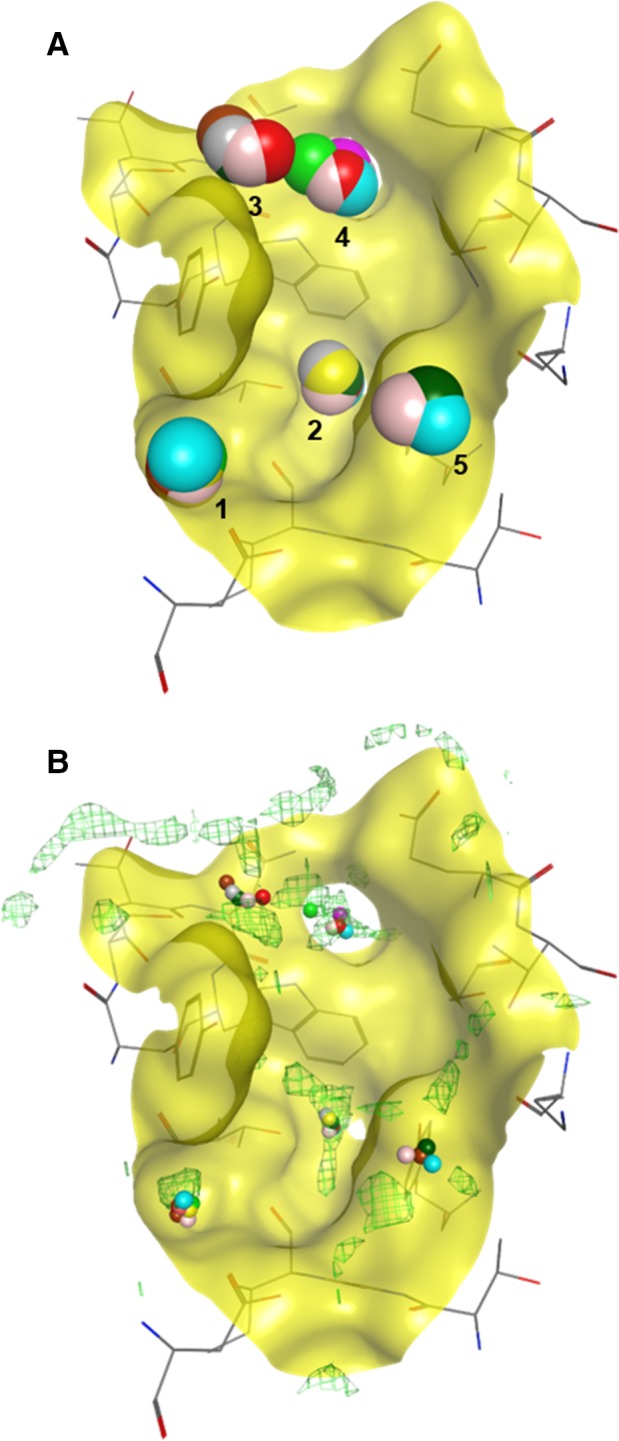
Analysis of hydration sites in the binding site of NRP1‐b1. (A) Superposition of nine high‐resolution X‐ray crystal structures (10 chains) (PDB entries: 4RN5, 2QQI, 1KEX, 5JGI, 5J1X, 5JGQ, 5IYY, 6JHK, 5IJR). 5 sites were identified and 4 or more structures show a water molecule conserved in that position. (B) Overlay of superposed structures showing conserved water molecules with the protein hydration prediction map. The computational model correctly predicted four sites where conserved water molecules were found.

The polypeptide chains were aligned to allow the visualisation of the crystallised water molecules in the binding site of NRP1‐b1. A water molecule was considered ‘conserved’ when observed in at least four out of ten superimposed molecules from the crystal structures (see methods for details). In total, five localised areas, labelled 1**–**5, on the molecular surface of the NRP1‐b1 ligand‐binding site were identified as containing the conserved water molecules (Fig. 8A). It was very encouraging to see that the computational hydration prediction using the MOE implementation of 3D‐RISM theory performed well with a structure of a lower resolution and this correlated with the experimentally derived solvent positions based on the X‐ray diffraction data. The 3D‐RISM predicted the location of four out of five experimentally determined conserved water molecules. The water molecule at site 5, near the surface of the NRP1‐b1 domain, was the only water molecule whose position was not predicted by this hydration analysis (Fig. 8). Free‐energy mapping suggests which water molecules are more stable and better targets for displacements in drug design (Table 4). In the apo NRP1‐b1 structure (1KEX), in addition to the water molecules corresponding to sites 1**–**4, crystallised waters are found at the sites commonly occupied by the carboxyl and guanidine groups of the arginine‐based compounds, as is also true for the prediction model. The calculated free energies for these two areas (−1.4 kcal·mol^−1^ and 0.12 kcal·mol^−1^ for the carboxylic acid site and guanidine site, respectively) indicate that these are stable waters, and that their replacement would be favourable.

**Table 4:** Characterisation of water clusters in NRP1‐b1

Cluster number	Free energy (kcal·mol^−1^)
1	1.30
2	1.15
3	−0.80
4	2.16
5	Nd
COO‐site (seen only in apo structure)	−1.40
Guanidine site (seen only in apo structure)	0.12

Sites 1 and 2 are occupied by water molecules in 9 out of 10 analysed structures. These are deep areas in the binding site, which could be classified as sub‐pockets, and could be targeted for replacement in drug design with the aim of enhancing affinity. The water molecule in site 3 is present in five structures. This molecule establishes a hydrogen bond with the side chain of Ser298 and is present in both apo‐ and ligand‐bound structures. Calculated free energy of site 3 (−0.8 kcal·mol^−1^) suggests that this cluster could also be target for displacement by a ligand's functional group. In fact, in the crystal structure of EG0229 (PDB code: 3I97), this site is occupied by a sulphonamide group. The conserved water molecule in site 4 is engaged in a hydrogen‐bond interaction with the nitrogen atom on the Trp301. This is observed in six structures. Additionally, this molecule is also seen engaging in hydrogen bonds with E348. In the structures with ligands **R4–R8**, the water molecule in site 4 additionally forms a hydrogen bond with the oxygen atom of the arginine analogue carboxyl group. However, in the structure of **R9**‐bound NRP1, the conserved water molecule in site 4 has been replaced by an oxygen atom of the **R9** carboxylate. As mentioned above, **R9** shows very weak binding affinity to the NRP1‐b1 domain, which can be interpreted by the loss of favourable direct hydrogen bonds of **R9** to the polypeptide chain together with a disruption of a water network caused by the displacement of the water molecule in site 4. This carboxyl/oxygen–water–Trp301/nitrogen interaction might be a key network in the binding of ligands to NRP1‐b1. We suggest this water molecule, which corresponds to the highest free‐energy site (2.16 kcal·mol^−1^), should be included in docking studies for drug design, and used as a bridge between the ligand and the protein instead of attempting to displace it or replace it with functional groups. The last conserved water molecule observed was in site 5, found in only four structures, and located near the protein surface, with a possible hydrogen bond to Tyr353. Since it is superficial water, we would not consider it significant for drug design.

### Implications for structure‐based drug design

We studied a known binding site in the NRP1‐b1 domain. To disrupt ligand binding to this domain, a protein–protein interaction, with a potent small molecule, it was important to perform an in‐depth study of the architecture and the solvation map of the target site. NRP1 shows a well‐defined arginine‐binding site where the guanidine part can establish a bidentate hydrogen‐bond interaction with the side chain of Asp320, and the C‐terminal carboxyl group can establish hydrogen bonds with the side chain oxygen atoms from Thr349 and Ser346. Although these interactions are conserved when small molecules containing a *N*α‐substituted arginine are bound to NRP1‐b1, the arginine analogues examined in this study exhibited varied affinities for the receptor. Both molecules with the highest affinity (**R5** and **R7**) contained carbamate group.

We obtained the crystal structures of six arginine analogues bound to NRP1‐b1 and analysed these alongside other publicly available structures in the PDB. The binding site of NRP1‐b1 shows little main chain and/or side chain flexibility, with only Tyr297 and Asp320 showing significant variations, which are dependent on the ligand bound. Tyr297 has been previously shown as required for VEGF binding, with NRP1Y297A/Y297A homozygous knock‐in mice exhibiting reduced VEGF‐induced angiogenesis and tumourigenesis 40. The availability of a closely related set of protein–ligand structures at high resolution provided a rare opportunity to test the water prediction capabilities of the software. Our computational prediction of the NRP1‐b1 binding site hydration showed good correlation with the location of the observed crystallographic waters. Especially important are the insights gained into the stability of the individual waters in the binding sites and whether they are likely to be displaced upon ligand (inhibitor) binding. Structural water molecules were found reproducibly within five buried subpockets in the immediate vicinity of the NRP1‐b1 ligand‐binding site; four of these sites were highly conserved with the fifth, less conserved one, positioned near the protein surface. We propose that displacement of these waters by small molecules may add to their binding affinity, with the exception of the conserved water molecule in site 4 which should in turn be retained in the binding site during docking studies. Displacement of this water molecule resulted in a much weaker ligand, as demonstrated by R9 analogue. Our analysis highlights the importance of considering structural water molecules in structure‐based drug design. When high‐resolution crystal structures are not available to determine conserved water molecules, simple computational methods may be used as an alternative.

## Experimental procedures

### Materials

Arginine analogues were obtained from commercial sources: **R1**,** R2**,** R8** (Sigma, Haverhill, UK), **R3**,** R7**,** R9** (AK Scientific), **R4**,** R5**,** R6** (Alfa Aesar, Heysham, UK), and used without further purification. All experiments were conducted using commercial reagents and standard procedures, unless stated otherwise.

### Computational prediction of conserved water molecules and molecular modelling

Molecular modelling was performed using the software package MOE (Molecular Operating Environment, 2014.09; Chemical Computing Group Inc., 1010 Sherbooke St. West, Suite #910, Montreal, QC, Canada, H3A 2R7, 2015).

#### Preparation of the input file

The structure used for the prediction of binding site hydration was PDB code: 3I97. Water and co‐crystallised ligands were removed from the structure. An arginine residue was modelled from the coordinates of EG00229 (ligand id: 8dr, PDB code: 3I97) and used to define the boundaries of the binding site. Hydrogen atoms were added to the structure by selecting the function ‘add hydrogen’ in MOE before the calculations were performed. The binding site for solvent prediction was defined by all atoms included in a 10 Å proximity from the ligand, extended by residues of those atoms.

#### Solvent analysis

Binding site hydration prediction was carried out using the ‘Solvent Analysis’ function in MOE. The application uses the three‐dimensional reference interaction site model (3D‐RISM) method 37, 41, 42 to analyse the role of solvent in proteins. It computes a time‐averaged distribution of water H and O densities, and free‐energy maps for analysing solvent stability and solvation contributions to binding free energy. The parameters used are as follows: the dimension of the grid spacing was 0.35 Å; a distance of 7 Å was set for the boundary box where atoms are extended; the convergence or precision of 3D‐RISM was setup to ‘tight’; the NDIIS (the Number of copies of Direct Inversion in the Iterative Subspace) was set to 5. This value balances the memory used and the convergence rate. The number of copies (N) of the various 3D grids retained in memory for accelerating convergence by extrapolation. Smaller values use less memory but slow down the convergence rate. A detailed description of these parameters can be found in the references 43.

#### Analysis of ordered water molecules

The SAS tool (Sequence annotated by Structure) 44 from the EMBL‐EBI website was used to retrieve all the related PDBs using 3I97 (NRP1‐b1) as a template. Only PDBs with a resolution better than 2.1 Å and sequence identity higher than 90% were retained for analysis. This resulted in three PDBs, which are 4RN5, 2QQI and 1KEX. In cases where the X‐ray crystal structure had more than one chain, both chains were analysed. Combined with the six structures obtained in‐house, a total of 9 PDBs or 10 chains were analysed. Among these structures, 2QQI and 1KEX are in the apo form.

#### Definition of binding site and conserved water molecule

The protein binding site was defined by a 5 Å proximity of all the ligand atoms present. Only the water molecules observed in the binding site were analysed. A water molecule is considered ‘conserved’ when observed in the same position of at least four superimposed crystal structures.

### Surface plasmon resonance

Surface plasmon resonance experiments were performed using a Biacore 4000 instrument at a constant temperature of 25 °C. Sensor chips, buffer stock solutions and immobilisation reagents were purchased from GE Healthcare.

#### Chip preparation

PBS containing 0.05% surfactant P20 was used as the running buffer during immobilisation. NRP1‐b1 was immobilised onto a CM5 chip using random amine coupling. The four flow cells were treated in the same way to optimise throughput. In summary, immobilisation spots 1 and 2 were activated with the coupling reagents, 1‐ethyl‐3‐(3‐dimethylaminopropyl)carbodiimide and *N*‐hydroxysuccinimide for 10 min. NRP1‐b1 at a concentration of 20 μg·mL^−1^ in 10 mm sodium acetate pH 5 was injected onto the surface for 10 and 5 min in spots 1 and 2, respectively, to generate surfaces with high and low density. The immobilisation levels ranged from 3487 to 3652 resonance units (RU) on spot 1 and from 1275 to 1756 RU on spot 2. Spot 3 was left unmodified and used as a reference.

#### Equilibrium affinity measurements

PBS containing 0.05% surfactant P20 was used as the running buffer and sample dilution buffer throughout these experiments. Dose‐responses were obtained using a two‐fold sample dilution, generating eight data points for the concentration range, and using an injection time of 60 s. Surface regeneration between injections was not necessary, but a wash step with 1M NaCl was included after injection of the highest concentration sample for each compound. All compounds were initially tested at a wide dilution range from 0.6 to 300 μm. Dilution series were then optimised for three compounds to obtain a more accurate measurements and dissociation constants. **R1** and **R9** were tested at 12**–**1500 μm, and **R5** was tested at 0.16**–**20 μm. Binding responses to high‐ and low‐density surfaces were processed independently and the average ± SD is presented. *K*_*D*_s reported are derived from steady‐state binding responses assuming a 1 : 1 interaction and therefore correspond to the equilibrium binding affinity of the compounds.

### Protein purification and crystallisation

NRP1‐b1 was expressed in *E. coli* strain Rosetta‐gami2‐(DE3)pLysS (Novagen) and purified as previously described 19. The protein was concentrated to 9**–**10 mg·mL^−1^ and 10 μL was mixed with 1 μL ligand at 10–100 mm in water. The complex was crystallised in a 1 : 1 volume mixture with 10**–**30% w/v PEG 3350, and 0.2 m ammonium chloride at 16 °C using hanging drop vapour diffusion method and micro‐seeding with 1/10 dilution of seeds of apo‐NRP1‐b1 crystals. Seeds were prepared according to Hampton Research's seeding kit. Crystals appeared within 2**–**3 days with needle morphology. A single crystal was transferred to a solution containing the crystallisation condition plus 20 % v/v ethylene glycol and was afterwards flash‐frozen in liquid nitrogen.

### X‐ray Crystallography

X‐ray diffraction data were collected at 100 K on beamline I04 at the Diamond Light Source, Didcot UK. All data sets were processed with xia2‐3d automated software system 45, 46, 47, 48, 49. Molecular replacement solutions were obtained by Phaser 50 using an apo NRP1‐b1 domain (PDB code 1KEX) as the search model. The refinement was carried out by Refmac5 51. The dictionary files for the fragments were generated using the acedgr programme from ccp4 software suite 52. Iterative rounds of building and refinement were carried out in COOT 53 and Refmac5. TLS (Translation/Libration/Screw) groups were generated automatically by Refmac5. Data collection and refinement statistics for all structures can be found in Table 1.

### Cell‐Free bt‐VEGF‐A_165_‐binding assay

The assay was run as previously described 19. The 96‐well plates were precoated with NRP1‐b1 protein at 3 μg·mL^−1^ overnight at 4 °C. On the following day, the plates were treated with blocking buffer (PBS containing 1% BSA) and washed three times with wash buffer (PBS containing 0.1% Tween‐20). The various concentrations of compounds diluted in PBS containing 1% DMSO were added, followed by addition of 0.25 nm bt‐VEGF‐A165. After 2 h of incubation at room temperature, the plates were washed three times with wash buffer. The bt‐VEGF‐A165 bound to NRP1‐b1 was detected by streptavidin‐horseradish peroxidase conjugates and the enzyme substrate and measured using a Tecan Genios plate reader at 450 nm absorbance with a reference wavelength at 595 nm. Non‐specific binding was determined in the absence of NRP1‐b1‐coated wells of the plates and subtracted from the readings obtained in the presence of NRP1‐b1. GraphPad Prism was used to carry out a non‐linear regression analysis and to generate inhibition curve and IC50 value. IC50 values for the particular ligand were obtained based on the three separate experiments.

## Conflict of interest

DS, IZ, PF and SD were consultants for Magnus Life Science.

## Author contributions

CF, RR and TY carried out the X‐ray crystallography; AT assisted with protein preparation. EC did the computational work. JH and DS selected the compounds for analysis. AOL carried out VEGF displacement assays. FM performed the SPR experiments, analysed the results and wrote the paper. DS, IZ, PF and SD supervised the research team and edited the paper.
